# Clustering of cancer data based on Stiefel manifold for multiple views

**DOI:** 10.1186/s12859-021-04195-4

**Published:** 2021-05-25

**Authors:** Jing Tian, Jianping Zhao, Chunhou Zheng

**Affiliations:** 1 College of Mathematics and System Sciences, Xinjiang University, Urumqi, China; 2 School of Computer Science and Technology, Anhui University, Hefei, China

**Keywords:** Stiefel manifold, Multi-view clustering, Cancer data, Optimization model, Linear search algorithm

## Abstract

**Background:**

In recent years, various sequencing techniques have been used to collect biomedical omics datasets. It is usually possible to obtain multiple types of omics data from a single patient sample. Clustering of omics data plays an indispensable role in biological and medical research, and it is helpful to reveal data structures from multiple collections. Nevertheless, clustering of omics data consists of many challenges. The primary challenges in omics data analysis come from high dimension of data and small size of sample. Therefore, it is difficult to find a suitable integration method for structural analysis of multiple datasets.

**Results:**

In this paper, a multi-view clustering based on Stiefel manifold method (MCSM) is proposed. The MCSM method comprises three core steps. Firstly, we established a binary optimization model for the simultaneous clustering problem. Secondly, we solved the optimization problem by linear search algorithm based on Stiefel manifold. Finally, we integrated the clustering results obtained from three omics by using k-nearest neighbor method. We applied this approach to four cancer datasets on TCGA. The result shows that our method is superior to several state-of-art methods, which depends on the hypothesis that the underlying omics cluster class is the same.

**Conclusion:**

Particularly, our approach has better performance than compared approaches when the underlying clusters are inconsistent. For patients with different subtypes, both consistent and differential clusters can be identified at the same time.

## Introduction

One of the challenges of cancer treatment is how to identify tumor subtypes, which can help to provide patients with specific treatment. Meanwhile, with the continuous development of all kinds of sequencing technologies, a lot of high flux data have been produced [1]. For cancer subtypes identification, integration of different types of omics data to unravel the molecular mechanism of complex diseases becomes more and more important [2]. On the one hand, multiple omics data of different subtypes of cancer provided more detailed information. On the other hand, it made data analysis more complicated. Different levels of multiple omics data often show different types, they have different correlation structure statistical properties and expressions [3]. In addition, the same tumor specimens from different levels of data are also unlikely to be independent. Therefore, how to reasonably integrate the multiple omics data to accurately predict cancer subtypes becomes a challenging and interesting research [4].

Due to the high dimensionality of data, we usually need to take a series of dimensionality reduction measures. However, some unsupervised approaches such as KCCA 5, KPCA [6], ISOMAP [7], the projection is only optimal at preserving the variance of the data or preserving the direction of the search. The two processes of reduction and clustering are completely independent. Solving the optimization problem on Stiefel manifold, it can be found directly in the lower dimensional representation of the feasible solution. It is worth mentioning that the noise in the original data can be reduced effectively by manifold methods. The literature [8] finds that when solving the optimization problem on Stiefel manifold, it can be more simple and quickly reach an almost medium precision.

Recently, many strategies for integrating multi-omics data have emerged. Their objectives are to understand the inter-relationships between different omics, and explore the relationship between omics data and subtypes [9, 10] . For example, the methods of biclustering aim to find the internal similar structure of high-dimensional data, and can cluster samples and features simultaneously. They have good performances in many ways, but they have a high time complexity [11, 12]. Similarity Network Fusion (SNF) method [13] constructed the similarity network for each data type, and then used the iterative method to fuse them into a similar network. The final clusters are obtained by spectral clustering of fusion networks. Some multi-view clustering methods based on spectral clustering have also been proposed [9, 14, 15]. They used different integration methods to combine the spectral clustering results from a single view. The Affinity Aggregation for Spectral Clustering (AASC) algorithm [14] introduced weights in the spectral clustering of each view, and then added them together to optimize the weights in the calculation.

However, these methods were put forward based on a basic hypothesis that the underlying omics clusters are the same. In actual situation, there are inconsistent clusters [10]. In the process of integrated clustering, data clustering was carried out for each view and cluster alignment was carried out for different views, which could handle this situation [9, 15, 16]. However, the method [9] tended to obtain the local optimal as described above, and the methods [15, 16] relax excessively the original multi-view point specific tangent condition, so that the information of each viewpoint may be lost. In the paper [17], the authors proposed the Multi-View Clustering using Manifold Optimization (MVCMO) method considered the diversity of the cluster. Consistent clusters and different clusters can be identified in each group. This method can effectively identify the cluster of differences, and this theory is also used in our method.

In order to improve the algorithm stability of MVCMO [17], we introduce the "Heat Kernel" to measure similarity between patients. And we use Backtracking Line Search to find the optimal solution more accurately. In this study, we propose a Multi-view Clustering based on Stiefel Manifold (MCSM) method for multi-view clustering problems with potential clusters. Firstly, we introduce a "Heat Kernel" to measure similarity. The patient-patient similarity network is constructed using k-nearest neighbor (KNN) method. Then we establish a binary optimization model for the simultaneous clustering problem. The solving process of the objective optimization problem is divided into three steps. First, we project our target function onto each of Stiefel manifold's tangent vector Spaces. Second, we do Backtracking Line Search on Stiefel manifold for the objective problem. Third, we retract the found points to the manifold with singular value decomposition. Finally, the KNN method is used for integrating the obtained clusters from three omics to get the final result. The proposed MCSM method has two highlights. One is that it combines the two processes of reduction and solution optimization,which preserves as much data information of each sample as possible. The other is that it can identify the cluster effectively when the underlying clusters are different. We experiment on simulated datasets to see the algorithm's performance when there are potential clusters. The experimental results on simulated datasets and several multiple omics datasets from TCGA show that our method has better performance than state-of-art methods.

## Datasets and methods

**Fig. 1:**
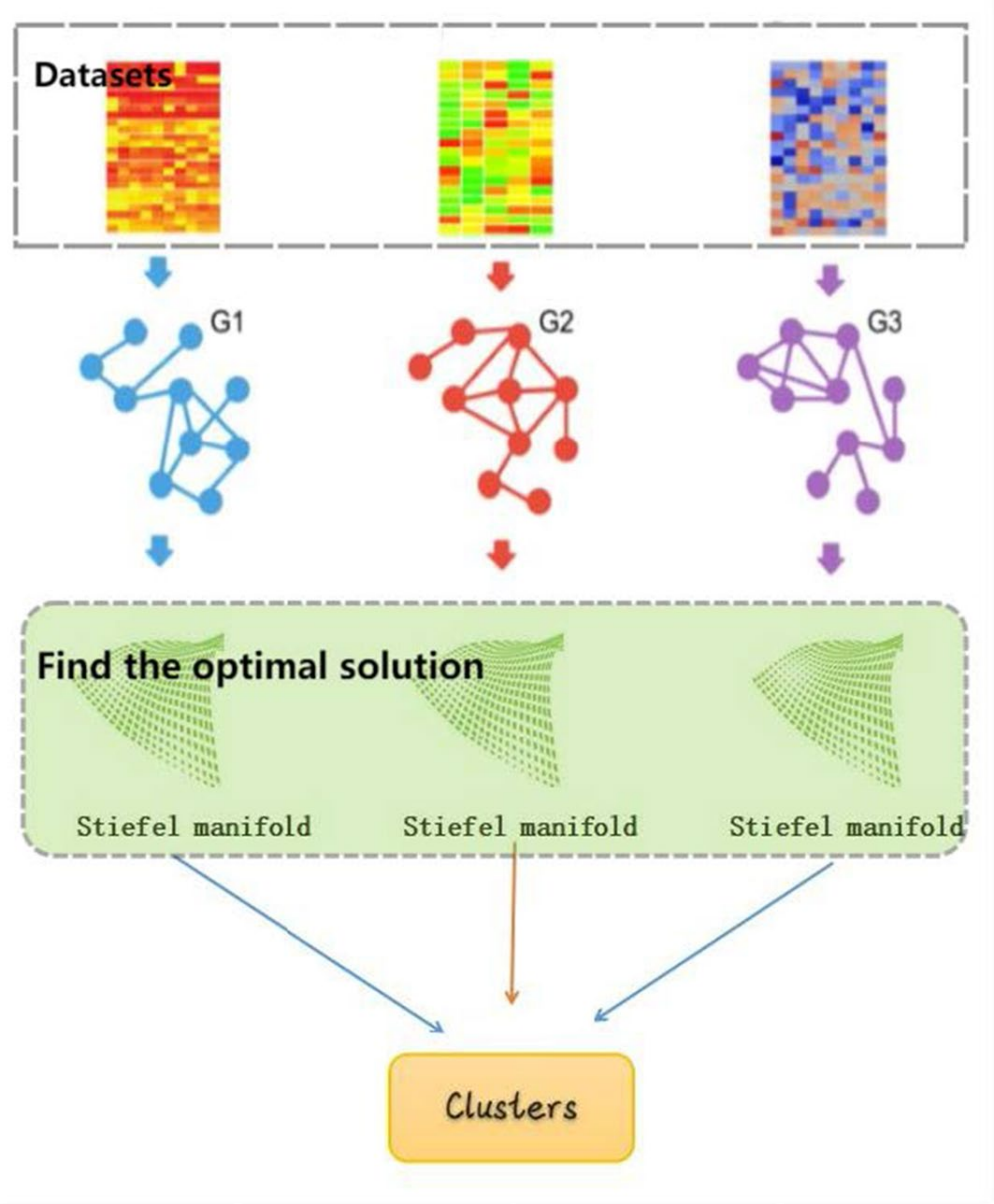
The process of MCSM method

The overall design of our method is illustrated in Fig. 1.

### Datasets and preprocessing

In this paper, we selected four cancer datasets in the TCGA for experiment, including gene expression data, miRNA expression data and DNA methylation data from samples of cancer patients. The cancer datasets include glioblastoma multiforme (GBM) with 215 samples, breast invasive carcinoma (BIC) with 105 samples, Skim Cutaneous Melanoma (SKCM) with 439 samples and Acute Myeloid Leukemia (AML) with 96 samples.

Firstly, if the data of a patient loses more than 20% in any data type, the patient will be deleted. Secondly, if the missing value of a feature in all patients exceeds 20%, it will be filtered out. Thirdly, the K-nearest-neighbor method is adopted to fill in missing data. We need to determine k according to the size of the sample. In our experiment, we set k = 20.

Fourthly, we log transform the data set to make it more stable. Finally, each feature is normalized in the constructed network to make it have a standard normal distribution. We performed the following normalization for each data type:

1 $$\widehat{f}=\frac{f-E(f)}{\sqrt{Var(f)}} .$$

where *f* is the characteristic of sample data,$\widehat{f}$ is the corresponding characteristic after normalization of *f*, E (*f*) and Var (*f*) represent the sample mean and sample variance respectively.

### Construction of the patient-to-patient similarity graph

Denoted ${\{{\mathrm{X}}^{\mathrm{m}}\}}_{\mathrm{m}=1}^{\mathrm{M}}$ as multi-view data from N patient samples, which has m data type in total. Each ${\mathrm{X}}^{\mathrm{m}}$ is a matrix of ${\mathrm{p}}_{\mathrm{m}}\times \mathrm{N}$, then a similar network graph ${\mathrm{G}}^{\mathrm{m}}$ is constructed to reflect the neighborhood relationship between the samples.

In the similar network of  the type m,${\mathrm{G}}^{\mathrm{m}}=({\mathrm{V}}^{\mathrm{m}},{\mathrm{E}}^{\mathrm{m}} ,{\mathrm{W}}^{\mathrm{m}} )$, ${\mathrm{V}}^{\mathrm{m}}$ is vertex set, ${\mathrm{E}}^{\mathrm{m}}$ is edge set, and ${\mathrm{W}}^{\mathrm{m}}$ is adjacency matrix. The adjacency matrix of ${\mathrm{W}}^{\mathrm{m}}$ in graph ${\mathrm{G}}^{\mathrm{m}}$ is a symmetric matrix.

In this paper, “Heat Kernel” is used to measure the similarity between samples [18]. The basic form is a Gaussian function with “t”. It has linear complexity and robustness that is not sensitive to small changes.

2 $${\mathrm{S}}_{\mathrm{ij}}^{\mathrm{m}}=\mathrm{exp}\left(-\frac{{\Vert {\mathrm{x}}_{\mathrm{i}}^{\mathrm{m}}-{\mathrm{x}}_{\mathrm{j}}^{\mathrm{m}}\Vert }^{2}}{2{\mathrm{t}}^{2}}\right),\mathrm{i}=1\dots ,\mathrm{N},\mathrm{j}=1\dots ,\mathrm{N}.$$

Next, we construct the K-nearest neighbor graph based on the similarity matrix ${\mathrm{S}}^{\mathrm{m}}$. If the vertex has an edge between ${\mathrm{v}}_{\mathrm{i}}$ and ${\mathrm{v}}_{\mathrm{j}}$, then ${\mathrm{W}}_{\mathrm{ij}}^{\mathrm{m}}$ represents the edge weight, otherwise 0.

3 $${\text{W}}_{{{\text{ij}}}}^{{\text{m}}} = \left\{ {\begin{array}{*{20}l} {{\text{S}}_{{{\text{ij}}}}^{{\text{m}}} ,} \hfill & {{\text{v}}_{{\text{j}}} \in {\text{N}}_{{\text{i }}} , } \hfill \\ {0,} \hfill & {otherwise.} \hfill \\ \end{array} } \right.$$

here ${\mathrm{N}}_{\mathrm{i}}$ is the neighborhood of ${\mathrm{v}}_{\mathrm{i}}$ (including ${\mathrm{v}}_{\mathrm{i}}$), ${\mathrm{N}}_{\mathrm{i}}$ with size k, and the number of k usually depends on the size of the sample. Essentially, we assume that local similarity is more reliable than remote similarity. This is a modest assumption, and it is widely used by other manifold learning algorithms [18].

### Construction of objective optimize problem

The objective optimize problem of the spectral clustering method is:

4 $$\begin{aligned} & \mathop {\min }\limits_{{{\text{U}}_{{\text{m}}} \in {\text{R}}^{{{\text{N}} \times {\text{k}}}} }} \,{\text{trace}}\,\left( {{\text{U}}_{{\text{m}}}^{{\text{T}}} {\text{L}}_{{\text{m}}} {\text{U}}_{{\text{m}}} } \right) \\ & {\text{s}}.{\text{t}}.\quad {\text{U}}_{{\text{m}}}^{{\text{T}}} {\text{U}}_{{\text{m}}} = {\text{I}}_{{\text{K}}} . \\ \end{aligned}$$

Here, the ${L}_{m}=({D}_{m}-{A}_{m})$. The ${A}_{m}$ is the corresponding adjacency matrix of similar network ${\mathrm{G}}^{\mathrm{m}}$, and ${D}_{m}$ is the diagonal matrix constructed using the degree of all the nodes in the mth network.

Then, used ${U}_{m}$ for K-means and find its minimum k eigenvectors in order to obtain the clustering labels.

Based on the spectral clustering, [15] proposed a multi-view network clustering method. Its objective optimize problem is:

5 $$\begin{aligned} & \min \mathop \sum \limits_{{{\text{m}} = 1}}^{{\text{M}}} \mathop \sum \limits_{{{\text{k}} = 1}}^{{\text{K}}} \frac{{\left( {{\text{S}}_{{,{\text{k}}}}^{{\text{m}}} } \right)^{{\text{T}}} \left( {{\text{D}}_{{\text{m}}} - {\text{A}}_{{\text{m}}} } \right)\left( {{\text{S}}_{{,{\text{k}}}}^{{\text{m}}} } \right)}}{{\left( {{\text{S}}_{{,{\text{k}}}}^{{\text{m}}} } \right)^{{\text{T}}} \left( {{\text{S}}_{{,{\text{k}}}}^{{\text{m}}} } \right)}} -\upbeta \mathop \sum \limits_{{{\text{l}} \ne {\text{m}}}} \mathop \sum \limits_{{{\text{k}} = 1}}^{{\text{K}}} \frac{{\left( {{\text{S}}_{{,{\text{k}}}}^{{\text{m}}} } \right)^{{\text{T}}} \left( {{\text{S}}_{{,{\text{k}}}}^{{\text{l}}} } \right)}}{{{\text{S}}_{{,{\text{k}}2}}^{{\text{m}}} {\text{S}}_{{,{\text{k}}2}}^{{\text{l}}} }} \\ & {\text{s}}.{\text{t}}.\,\,{\text{S}}_{{,{\text{k}}}}^{{\text{m}}} \in \left\{ {0,1} \right\},{\text{i}} = 1 \ldots ,{\text{N}};{\text{m}} = 1 \ldots ,{\text{M}};{\text{k}} = 1 \ldots ,{\text{k}}; \\ & \mathop \sum \limits_{{{\text{k}} = 1}}^{{\text{K}}} {\text{S}}_{{{\text{i}},{\text{k}}}}^{{\text{m}}} = 1,{ }\quad {\text{for }}\quad {\text{ m}} = 1 \ldots ,{\text{M}}. \\ \end{aligned}$$

The binary optimization problem cannot be solved in polynomial time. So, the objective function of multi-view spectral clustering can be constructed as follows:

6 $$\begin{aligned} & \mathop {\min }\limits_{{{\text{U}}_{{\text{m}}} \in {\text{R}}^{{{\text{N}} \times {\text{k}}}} }} \,{\text{trace}}\,\left( {{\text{U}}^{{\text{T}}} {\text{LU}}} \right) \\ & {\text{s}}.{\text{t}}.\quad {\text{U}}^{{\text{T}}} {\text{U}} = {\text{I}}_{{\text{K}}} \\ \end{aligned}$$

where $$\mathrm{L}=\left(\begin{array}{ccc}{\mathrm{L}}_{1}& \cdots & 0\\ \vdots & \ddots & \vdots \\ 0& \cdots & {\mathrm{L}}_{\mathrm{m}}\end{array}\right)-\upbeta \left(\begin{array}{ccc}0& \cdots & {\mathrm{I}}_{\mathrm{n}}\\ \vdots & \ddots & \vdots \\ {\mathrm{I}}_{\mathrm{n}}& \cdots & 0\end{array}\right),\mathrm{U}=\left(\genfrac{}{}{0pt}{}{\begin{array}{c}{\mathrm{U}}_{1}\\ {\mathrm{U}}_{2}\end{array}}{\begin{array}{c}\vdots \\ {\mathrm{U}}_{3}\end{array}}\right)$$.

$\beta$ is used to balance the weight parameters between the network and within the network. If we have abundant prior knowledge, we can set it according to prior information. Otherwise, when building a network, we can try to establish a connection at the same level (e.g. similar connection densities) and set it directly to 1. In our experiment, we set $\beta =1$ directly.

However, the optimization problem (6) combines the information of all networks together and will loss the information in each network. The proposed MVSM method still follows the original objective function of multi-view spectral clustering and the construction of Laplace matrix [17].

The objective optimization problems to be solved are as follows:

7 $$\begin{aligned} & \mathop {\min }\limits_{{{\text{U}}_{{\text{m}}} \in {\text{R}}^{{{\text{N}} \times {\text{k}}}} }} {\text{trace}}\left( {{\text{U}}^{{\text{T}}} {\text{LU}}} \right) \\ & {\text{s}}.{\text{t}}.\quad {\text{U}}_{{\text{m}}}^{{\text{T}}} {\text{U}}_{{\text{m}}} = {\text{I}} \\ \end{aligned}$$

When we set ${\mathrm{U}}_{\mathrm{m}}=\frac{{\mathrm{S}}^{\mathrm{m}}}{{\Vert {\mathrm{S}}^{\mathrm{m}}\Vert }_{2}}$, the objective function ${\mathrm{U}}_{\mathrm{m}}^{\mathrm{T}}{\mathrm{U}}_{\mathrm{m}}={\mathrm{I}}_{\mathrm{K}}$ substitude as $\sum {\mathrm{s}}_{\mathrm{i},\mathrm{j}}^{\mathrm{N}}=1$. It transforms the constraints for each network into one equation.

### The solution of objective optimize problem

To solve the objective function (7), we project it onto the Stiefel manifold and solve it by  backtracking linear search. The process is roughly divided into three steps.

First, we project the target function $\mathrm{trace}\left({\mathrm{U}}^{\mathrm{T}}\mathrm{LU}\right)$ onto each of Stiefel manifold's tangent vector Spaces.

The tangent vector space of M is.

8 $${\mathrm{TM}}_{\mathrm{m}}=\left\{{\mathrm{U}}_{\mathrm{m}}\mathrm{B}+\left(\mathrm{I}-{{\mathrm{U}}_{\mathrm{m}}\mathrm{U}}_{\mathrm{m}}^{\mathrm{T}}\right)\mathrm{C}:\mathrm{B}=-{\mathrm{B}}^{\mathrm{T}},\mathrm{C}\in {\mathrm{R}}^{\mathrm{N}\times \mathrm{k}}\right\}.$$

here each Stiefel manifold ${\mathrm{M}}_{\mathrm{m}}={\mathrm{U}}_{\mathrm{m}}\in {\mathrm{R}}^{\mathrm{N}\times \mathrm{k}}:\mathrm{ s}.\mathrm{t}.{\mathrm{U}}_{\mathrm{m}}^{\mathrm{T}}{\mathrm{U}}_{\mathrm{m}}={\mathrm{I}}_{\mathrm{K}}$.

So, the negative gradient of the target function $\mathrm{trace}\left({\mathrm{U}}^{\mathrm{T}}\mathrm{LU}\right)$ can be expressed as:

9 $$\mathrm{Z}=-\nabla \mathrm{ trace}\left({\mathrm{U}}^{\mathrm{T}}\mathrm{LU}\right)=-\mathrm{LU}={\left({\mathrm{Z}}_{1}^{\mathrm{T}},{\mathrm{Z}}_{2}^{\mathrm{T}},\dots ,{\mathrm{Z}}_{\mathrm{m}}^{\mathrm{T}}\right)}^{\mathrm{T}}$$

where $Z_{m}$ represents the negative derivative of the objective function on the mth omic.

Then, we search the next point along the direction ${\upeta }_{\mathrm{m}}$ on each tangent vector space of the manifold. Where,

10 $${\upeta }_{\mathrm{m}}={\mathrm{Z}}_{\mathrm{m}}-\frac{1}{2}{\mathrm{U}}^{\mathrm{m}}\left({\left({\mathrm{U}}^{\mathrm{m}}\right)}^{\mathrm{T}}{\mathrm{U}}^{\mathrm{m}}+{\mathrm{Z}}_{\mathrm{m}}^{\mathrm{T}}{\mathrm{U}}^{\mathrm{m}}\right).$$

Second, we do Backtracking Line Search on Stiefel manifold for problem (9–10).

The purpose of line search is to find the smallest point of the target function in the search direction. However, it is time-consuming to find the accurate minimum point. The search direction is already approximate, so we just to find the minimum point approximation at a lower cost. Backtracking Line Search (BLS) is such a Line Search algorithm. The idea of the BLS algorithm is to set an initial step size ${\alpha }_{0}$ in the search direction. Then, if the step size is too large, we reduce the step size until it is appropriate.

Backtracking Line Search in the negative gradient direction of the objective function is as follow:

11 $$\begin{aligned} {\text{f}}\left( {{\text{U}} + \upalpha \upeta } \right) & \le {\text{f}}\left( {\text{U}} \right) + \upalpha {\text{cm}} \\ {\text{m}} & =\upeta ^{T} {\text{Z}} \\ \end{aligned}$$

where $\upeta$ is the current search direction, $\mathrm{\alpha }$ is the step size, and c is the control parameter, which needs to be manually verified according to the situation.

If the current U does not satisfy inequation (11), then a parameter $\uptau$ is required to adjust the step size:

12 $$\mathrm{\alpha }=\mathrm{\tau \alpha }$$

where the parameter $\uptau$ is controls the reduce search step size.

Third, we retract the found points to the manifold with singular value decomposition.

13 $$\mathrm{U}=\mathrm{W\Sigma }{\mathrm{U}}^{\mathrm{T}},\mathrm{U}={\mathrm{WU}}^{\mathrm{T}}.$$

After the manifold optimization process, we get the values of U. The whole process of our proposed method is summarized in Algorithm 1.
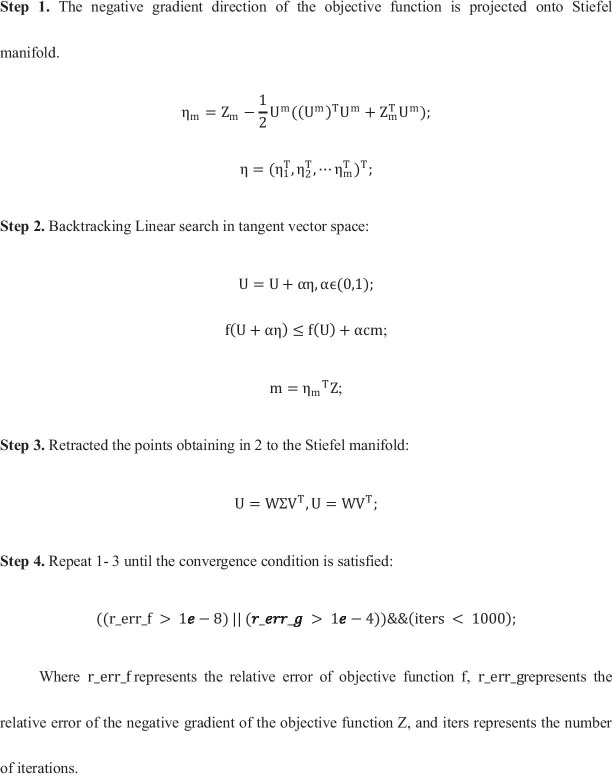


Here, we get the solution of the objective function, and then we perform k-means to cluster U and obtain the cluster labels ${\mathrm{C}}_{1},{\mathrm{C}}_{2},\dots ,{\mathrm{C}}_{\mathrm{k}}$. Finally, we integrate the clustering results obtained from three omics by using k-nearest neighbor method.

Remark: we set $\mathrm{c}=6,\uptau =0.1$ in our experiment. We will set out the reasons in Sect. 3.1.2.

## Results

In this section, we selected some methods from different perspectives to compare with MVSC methods. For the methods were proposed using network structure, we chose AASC algorithm [14], SNF method [13] and MVSC [15]. In particular, AASC and MVSC method can effectively identify different clusters. For the methods based on manifold, we chose MOCMO [17] and Grassmann manifold clustering method [18]. For the state-of-the-art methods, we chose MvNE algorithm [19].

### The selection of parameter

#### The number of clustering

When the clusters k is not known, we can select it according the value of silhouette [20] and RI coefficient. From the perspective of computational efficiency, Calinski  Harabaz score [21] is the highest. So Calinski  Harabaz score is more commonly used. Firstly, we did experiments with k equals 2–10. Then, we choose the  clustering number corresponding to the maximum Calinski Harabaz score. To compare MVSM to other methods, we set k as a known value.

#### The Backtracking Line Search parameters

There are three parameters in the Backtracking Line Search parameters, $\upeta ,\mathrm{ \alpha }$ and c. Where, $\upeta$ is the current search direction, $\mathrm{\alpha }$ is the step size, and c is the control parameter, which needs to be manually verified according to the situation. Firstly, we initialize $\mathrm{\alpha }=0.01$. During the experiment, it was found that if the value of c was too small, the step size would not be adjusted during the search process. However, if we want to adjust appropriately, then we need to set the parameter c according to the objective function value and gradient value of the initial point. Therefore, according to several data sets used in the experiment, we set $\mathrm{c}=6,\uptau =0.1$.

### Experimental results on simulated datasets

Here, we use the simulated datasets to verify that MVSM method is suitable for datasets with uneven distribution of underlying clusters.

Since these methods (AASC, SNF and MVSC) were proposed using network tools, we simulate the network structure firstly. Then, we generate the connections within the same cluster and different clusters. The probability of connections within a given cluster is greater than the probability of connections between clusters. For M omics networks, given the number of nodes N, these nodes are assigned to K clusters with different probabilities.

In order to see the influence of the connections between clusters change, we set the following four connection probability matrices:

$$\begin{aligned} {\text{P}}_{1} & = \frac{1}{{\text{N}}}\left( {\begin{array}{*{20}c} {16} & 0 & 0 \\ 0 & {18} & 0 \\ 0 & 0 & {17} \\ \end{array} } \right),{\text{ P}}_{2} = \frac{1}{{\text{N}}}\left( {\begin{array}{*{20}c} {16} & {0.4} & {0.6} \\ {0.4} & {18} & {0.55} \\ {0.6} & {0.55} & {17} \\ \end{array} } \right), \\ {\text{P}}_{3} & = \frac{1}{{\text{N}}}\left( {\begin{array}{*{20}c} {16} & {0.8} & {1.2} \\ {0.8} & {18} & {1.1} \\ {1.2} & {1.1} & {17} \\ \end{array} } \right),{\text{ P}}_{4} = \frac{1}{{\text{N}}}\left( {\begin{array}{*{20}c} {16} & {1.2} & {1.8} \\ {1.2} & {18} & {1.65} \\ {1.8} & {1.65} & {17} \\ \end{array} } \right). \\ \end{aligned}$$

The term (i,j) of the four matrices represents the connection probability between cluster i and cluster j. Each term $\left(\mathrm{i},\mathrm{i}\right)$ and each term $\left(\mathrm{i},\mathrm{j}\right),\mathrm{i}\ne \mathrm{j}$ represent the connections within and between clusters, the larger the value the term correspond to, the tighter the connection. N represents the number of nodes.

In order to see the performance of the method, we tested two settings. For each setting, we consider that the M omics of distribution is different.

Setting 1: M = 3, N = 150, cluster distribution: (50, 50, 50); (30, 90, 30); (40, 60, 50);

Setting 2: M = 6, N = 1000, cluster distribution: (300, 300, 400); (300, 300, 400); (400, 300, 300); (300, 350, 350); (300, 400, 300); (450, 250, 300).

We use the Rand index to evaluate the clustering performance, which is defined as:

$$\mathrm{RI}=\frac{\mathrm{TP}+\mathrm{FN}}{\mathrm{TP}+\mathrm{FP}+\mathrm{TN}+\mathrm{FN}} ,$$

'TP' is defined as the number of intersection nodes in the same cluster, which are also clustered in the same cluster, and other nodes are defined similarly.

On this basis, we obtain the rand index comparison of several methods:

For each setting, we run it 50 times and take the average of the results. Tables 1 and 2 show the mean RI when the underlying clusters are different for the two settings.We can see that all four methods with an average RI is close to 1 when the cluster sizes of different groups are the same. On the one hand, because both SNF and AASC set the underlying cluster to be the same, they cannot detect the difference between the different views. So the MVSC and our method have better performance, when the size of the underlying cluster is different. On the other hand, more information of the clusters can be kept in our method by using more strict relaxation of the binary variables. Form Tables 1 and 2, when the nodes of networks are different, our method has a  better performance than MVSC in both setups.

**Table 1:** Comparison of RI in different methods based on Setting 1

Method	${\mathrm{P}}_{1}$	${\mathrm{P}}_{2}$	${\mathrm{P}}_{3}$	${\mathrm{P}}_{4}$
AASC	0.73	0.73	0.73	0.73
SNF	0.68	0.67	0.67	0.67
MVSC	0.99	0.98	0.94	0.87
MCSM	1	0.99	0.94	0.97

**Table 2:** Comparison of RI in different methods based on Setting 2

Method	${\mathrm{P}}_{1}$	${\mathrm{P}}_{2}$	${\mathrm{P}}_{3}$	${\mathrm{P}}_{4}$
AASC	0.75	0.75	0.75	0.75
SNF	0.75	0.75	0.75	0.75
MVSC	0.93	0.93	0.93	0.93
MCSM	0.96	0.95	0.95	0.95

To further show the effective of our method, we also calculate the NMI coefficient on different omics. It is defined as follows:

$$\mathrm{NMI}(\mathrm{U},\mathrm{V})=\frac{2\mathrm{MI}(\mathrm{U},\mathrm{V})}{\mathrm{H}(\mathrm{U})+\mathrm{H}(\mathrm{V})}$$

where U and V represent the clusters according to clustering and real clusters, respectively. H(U), H(V) and MI(U, V) are defined as:

$$\begin{aligned} {\text{MI}}\left( {{\text{U}},{\text{V}}} \right) & = \mathop \sum \limits_{{{\text{i}} = 1}}^{{\text{C}}} \mathop \sum \limits_{{{\text{j}} = 1}}^{{\text{C}}} {\text{p}}_{{{\text{i}},{\text{j}}}} {\text{log}}\left( {\frac{{{\text{p}}_{{{\text{i}},{\text{j}}}} }}{{{\text{p}}_{{\text{i}}} \times {\text{p}}_{{\text{j}}} }}} \right) \\ {\text{H}}\left( {\text{U}} \right) & = - \mathop \sum \limits_{{{\text{i}} = 1}}^{{\text{C}}} {\text{p}}_{{\text{i}}} {\text{logp}}_{{\text{i}}} \\ {\text{H}}\left( {\text{V}} \right) & = - \mathop \sum \limits_{{{\text{j}} = 1}}^{{\text{C}}} {\text{p}}_{{\text{j}}} {\text{logp}}_{{\text{j}}} \\ \end{aligned}$$

${p}_{i}$ is the proportion of the number of cluster i to the total amount of sample.

For setting 2, we calculate NMI coefficients on each omics and show their contrast results in Table 3. It can be seen that the NMI coefficient of the MVSM method is higher than the contrast methods in all three omics.

**Table 3:** Comparison of NMI in different methods based on Setting 2

Method	${\mathrm{M}}_{1}$	${\mathrm{M}}_{2}$	${\mathrm{M}}_{3}$
AASC	0.84	0.64	0.90
SNF	0.74	0.49	0.88
MVSC	0.83	0.93	0.97
MCSM	0.84	0.97	0.97

### Experimental results on real datasets

In order to prove the effectiveness of our method on the real datasets. We apply our method on multiple omics datasets [22], analyze and compare our results with other advanced methods. Shown are final Cox survival P values for Glioblastoma Multiforme (GBM), breast Invasive carcinoma (BIC), Skim Cutaneous Melanoma (SKCM) and Acute Myeloid Leukemia (AML) in Table 4.

**Table 4:** Comparison of Cox survival p-values

Cancer type	SNF	Grassmann Cluster	MOCMO	AASC	MVSC	MvNE	MCSM
GBM(3)	0.0002	0.0043	0.0019	0.0022	0.00072	0.01113	**0.0001**
BIC(5)	0.0011	0.0002	**0.00016**	0.00015	0.0007	0.0061	0.0025
SKCM(4)	0.0001	0.19	0.00045	0.00016	0.00045	0.0098	**0.0001**
AML(5)	0.037	0.12	0.03	0.045	0.058	0.062	**0.019**

Bold values indicate the smallest Cox survival p-values on the different datasets

**Fig. 2:**
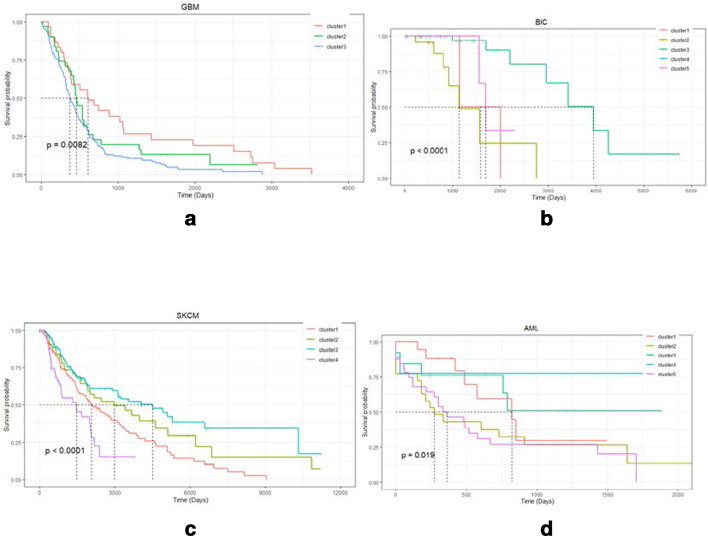
Survival plots for GBM, BIC, SKCM, and AML tumors

Shown are Kaplan Meier plots of the overall survival of integrative clusters for GBM (a), BIC (b), SKCM (c) and AML) (d) in Fig. 2.

It can be seen from the Table 4, in three of the four cancers datasets (GBM, BIC, SKCM and AML), our method obtains more significant differences than comparison methods in survival time. For BIC dataset, the insignificant difference may be due to the small cluster difference of the data itself. Survival plots for GBM, BIC, SKCM, and AML tumors are shown in Fig. 2. We can predict survival rate in a sample according plots in Fig. 2. In the prediction task, our method performed better than other methods.

### Convergence analysis

The proposed methodology can be divided into three parts, construction of Laplace matrix, process for solving optimization problems and iteration. The time complexity of each part of the algorithm is as follows:

*Construction of Laplace matrix* It has linear complexity.

*In the process of solving the optimization problem* The time complexity of matrix multiplication is $\mathrm{O}(2{\mathrm{Nk}}^{2})$.

*Iteration* Since we need to use SVD to retract the found solution back to the Stiefel manifold, then the matrix operation complexity is $\mathrm{O}({\mathrm{N}}^{3})$.

**Fig. 3:**
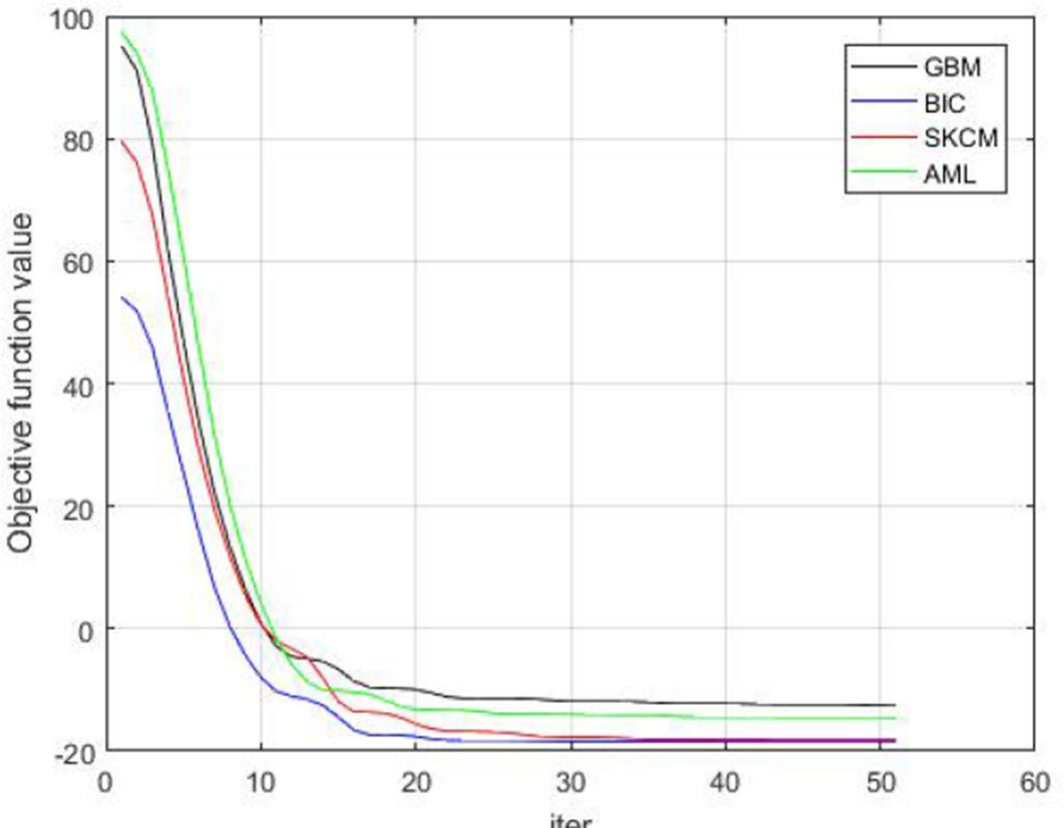
Convergence of the MVSM algorithm

Overall, the time complexity of MVSM method is much lower than that of MvNE method (O(nt(ij + jk + kl + lm))). From Fig. 3, it can be observed that for 20 iterations, there is a stable objective function value for all the datasets. It shows that our algorithm can find an appropriate solution with fewer iterations.

### A case study: comparison of established subtypes

In order to compare the results of our clustering with the established biological subtypes, we downloaded the clinical data of 215 GBMs from the cBio Cancer Genomis Portal (http://www.cbioportal.org/) at the Memorial Sloan-Kettering Cancer Center. For the GBM, there are four established subtypes defined by patients’ gene expression profiles, which are Classical, Mesenchymal, Neural and Proneural [23]. According to DNA methylation clustering, they [24] divided the subtypes into Glioma-CpG island methylator phenotype (G-CIMP) and Non-G-CIMP. The results of our method are compared with the established subtypes in Table 5. It shows that the clustering of our method is not just based on one data type, it takes into account both gene expression and DNA methylation information.

**Table 5:** Comparison of clusterings to established subtypes

Clusters	Gene expression subtypes				DNA methylation subtypes	
	Classical	Mesenchymal	Neural	Proneural	G-CIMP	Non-G-CIMP
Cluster1	0	0	1	13	16	14
Cluster2	2	25	2	2	0	31
Cluster3	55	41	31	24	2	152

For gene expression subtypes, it can be seen that cluster 1 mainly contains Proneural subtype, cluster 2 mainly contains Proneural subtype, and they have strong enrichment. However, for DNA methylation subtypes, G-CIMP subtypes are mainly distributed in cluster 1. If only the DNA methylation information is considered, cluster2 and cluster3 are likely to merge. So, we can conclude that it's important to consider both gene expression and DNA methylation information.

**Fig. 4:**
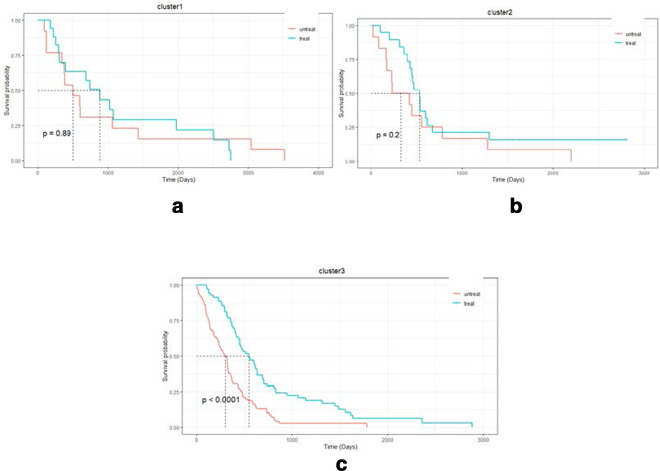
Survival analysis of GBM patients for treatment with TMZ

In order to further understand the biological significance of clusters, we investigated the response to temozolomide (TMZ) treatment of the GBMs. TMZ is an alkylation agent that causes incorrect pairing of thymine during DNA replication. In the GBM dataset, 105 patients were treated with TMZ. Figure 4 indicated that the TMZ-treated samples had different drug responses compared to the samples not treated with the drug. For different clusters, the degree of drug response of TMZ was also different. Compared with Cluster 1 and Cluster 2, patients in Cluster 3 had significantly increased survival time after treatment with TMZ (P value using Cox log-rank test = 0.0001), and this medication was also more meaningful. The results show that the clusters we obtained can be used as a reference for identifying the effectiveness of drugs.

## Conclusion

Multi-view data clustering is a hot topic in recent years. Recent work has focused on cases where the underlying clusters are consistent, and as we reviewed in the first section, several approaches have been proposed. When the underlying cluster is different, some methods are proposed to find different clusters. However, as we know, both consistent and differentiated clusters can exist at the same time. This leads us to study multi-view simultaneous clustering to find both consistent and different cluster data. In this paper, we propose a multi-view clustering model. On the basis of manifold optimization, the algorithm for formula optimization is proposed. Simulation results show that the performance of the proposed method is better than that of the existing algorithm under the same underlying cluster condition. We download the gene expression, miRNA expression and DNA methylation datasets of GBM, BIC, SKCM and AML from TCGA, and also carry out numerical experiments, showing that our method is superior to several comparison methods. In the future work, the cluster difference problem is still worth researching, and we will integrate other omics information such as gene mutation data.

## Data Availability

All the raw data are available at http://acgt.cs.tau.ac.il/multi_omic_benchmark/download.html (Nimrod et al., 2018), and all code scripts used are available at https://github.com/charley410/tianjing.
